# Enhanced Coloration Time of Electrochromic Device Using Integrated WO_3_@PEO Electrodes for Wearable Devices

**DOI:** 10.3390/bios13020194

**Published:** 2023-01-28

**Authors:** Haneul Kwon, Soohyun Kim, Mirim Ham, Yewon Park, Haekyoung Kim, Wonmok Lee, Hyunjung Lee

**Affiliations:** 1 School of Advanced Material Engineering, Kookmin University, Seoul 02707, Republic of Korea; 2 School of Materials Science and Engineering, Yeungnam University, Gyeongsan 38541, Republic of Korea; 3 Department of Chemistry, Sejong University, Seoul 05006, Republic of Korea

**Keywords:** electrochromic performance, tungsten trioxide (WO_3_), poly(ethylene oxide) (PEO), wearable devices, ion transport

## Abstract

Electrochromic technologies that exhibit low power consumption have been spotlighted recently. In particular, with the recent increase in demand for paper-like panel displays, faster coloration time has been focused on in researching electrochromic devices. Tungsten trioxide (WO_3_) has been widely used as an electrochromic material that exhibits excellent electrochromic performance with high thermal and mechanical stability. However, in a solid film-type WO_3_ layer, the coloration time was long due to its limited surface area and long diffusion paths of lithium ions (Li-ions). In this study, we attempted to fabricate a fibrous structure of WO_3_@poly(ethylene oxide) (PEO) composites through electrospinning. The fibrous and porous layer showed a faster coloration time due to a short Li-ion diffusion path. Additionally, PEO in fibers supports Li-ions being quickly transported into the WO_3_ particles through their high ionic conductivity. The optimized WO_3_@PEO fibrous structure showed 61.3 cm^2^/C of high coloration efficiency, 1.6s fast coloration time, and good cycle stability. Lastly, the electrochromic device was successfully fabricated on fabric using gel electrolytes and a conductive knitted fabric as a substrate and showed a comparable color change through a voltage change from −2.5 V to 1.5 V.

## 1. Introduction

An electrochromic device (ECD) is a device that can reversibly change its color when it undergoes a redox reaction. ECDs are expected to be widely used in various fields such as smart windows [1], rearview mirrors [2], and digital displays [3,4], which demand low power consumption, high coloration efficiency (η), stable reversibility, and a fast coloration time. At present, because of their low cost and small weight, they are increasingly being used in paper-like flat panel displays [3]. Electrochromic materials can be divided into organic materials (e.g., poly(3,4-ethylene dioxythiophene) and polyaniline) [5,6,7,8,9] and inorganic materials (e.g., tungsten trioxide (WO_3_), nickel oxide, and vanadium oxide) [10,11,12,13]. Although organic electrochromic materials have a relatively short coloration time (several seconds) and various color changes, their demerits, such as low reversibility, short lifetime, and a small voltage range, render them unsuitable for use in digital displays [5,7,9,11,14]. On the other hand, inorganic materials have many advantages, such as excellent η, good reversibility, long lifetime, high chemical stability, and low cost, although the coloration time is long (up to tens of seconds). Therefore, research to overcome this drawback of inorganic materials is ongoing [3,6].

WO_3_ is one of the widely used inorganic electrochromic materials with excellent electrochromic performance and high thermal and mechanical stability. The electrochromic reaction of WO_3_ is as follows [14]:

(1) $$WO_{3}\left(bleached\right)+x\left(M^{+}+e^{-}\right)\to M_{x}W_{1-x}^{6+}W_{x}^{5+}O_{3}\left(colored\right),$$

In this reaction, WO_3_ is in a bleached state when there is no applied voltage. When a negative voltage is applied, monovalent ions (H^+^, Li^+^, Na^+^, and K^+^) of the electrolyte penetrate the WO_3_ lattice, and W^6+^ is reduced to W^5+^. Consequently, the electrochromic layer becomes blue, a colored state. If positive voltage is applied to the electrochromic layer, W^5+^ is oxidized to W^6+^ and returns to the bleached state. Such electrochromic materials demand a fast coloration time, a high Δ*T*, and a high η for high performance. The conventional WO_3_ film has mainly been manufactured as a solid thin film on a conductive substrate. A film-type electrochromic layer usually has a long coloration time because of the long diffusion path [15,16]. The long diffusion path causes lithium-ions (Li-ions) to react only with the top layer, and the ions take considerable time to diffuse to the bottom. To reduce the coloration time, Zhao et al. fabricated a WO_3_ film with controllable crystallinity on an indium tin oxide (ITO) substrate by controlling the heat treatment rate [17]. The fabricated WO_3_ film had an amorphous phase at the top and a crystalline phase at the bottom. The amorphous WO_3_ phase was conducive to Li-ion transport, and hence, the film showed a high transmittance difference (Δ*T*) (72.5%), a fast coloration time (5.3 s), and a high η (80.5 cm^2^/C). While this dense film structure was readily fabricated using the magnetron sputtering method and its surface area was small, the Li-ion diffusion path was long [4,18]. Recent research on the fabrication of porous WO_3_ films has been focused on achieving a fast coloration time. The porous structure provides a large surface area for Li-ion reaction and reduces the diffusion path, increasing the number of active sites and decreasing the coloration time [4,19]. To fabricate a porous structure, a high-temperature process has usually been applied. Wang et al. synthesized a mesoporous WO_3_ film by using thermally decomposed WCl_6_ in a polymer composite [20]. Similarly, Kim et al. fabricated a mesoporous WO_3_ film by removing thermally carbonized WCl_6_ in a polymer composite using O_2_ plasma [21]. A mesoporous film prepared by a high-temperature process had a short coloration time and a high η. But in the high-temperature process, it was difficult to control the crystallinity of WO_3_, and the fabrication process was complex. Hence, it is necessary to solve these problems, such as the slow manufacturing speed, high process temperature, and high cost [12,22,23].

**Figure 1:**
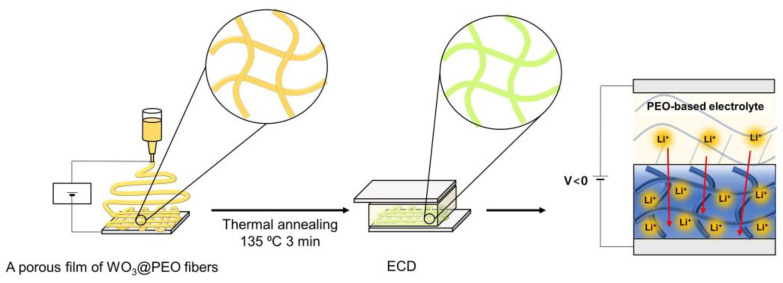
Schematic of the fabrication of the electrospun WO_3_@PEO-based electrochromic device.

Here, we prepared a porous WO_3_ electrochromic layer by fabricating a fibrous structure of WO_3_@poly(ethylene oxide) (PEO) composites (the composite is hereafter denoted by WO_3_@PEO) using a simple electrospinning method and a low process temperature (Figure 1). The porous film of WO_3_@PEO fibers had many active sites for Li-ions to react in comparison with a nonporous film, and it also had a shorter diffusion path, which resulted in a fast coloration time. Furthermore, owing to the use of PEO as a promoter of Li-ion transport, Li-ions could be expected to be easily transported through PEO into the WO_3_ particles. In particular, since a low-temperature process was employed, unexpected crystallization was prevented, and the fabrication cost of the porous WO_3_ layer was low [4,22,24]. In our research, the ratio and thickness of WO_3_ and PEO were adjusted to achieve an optimized porous structure, and we electrochemically characterized them. Then, we verified the cycle stability of the optimum condition of porous WO_3_@PEO fibers.

## 2. Materials and Methods

### 2.1. Materials

The dispersion of WO_3_ particles in ethanol (ECS-C1) was purchased from Adchro Inc. PEO with molecular weights (*M*_w_) of 100,000 and 600,000, anhydrous propylene carbonate (PC, 99.7%), and ferrocene (Fc, 98%) were purchased from Sigma-Aldrich Inc. (St. Louis, MO, USA). Lithium perchlorate anhydrous (LiClO_4_) was purchased from Wako Pure Chemical Corporation (Osaka, Japan), and anhydrous ethanol (99.9%) was purchased from Daejung Chemicals (Seoul, Korea) and Metals and used without additional purification. For assembling the ECD, we used indium-tin oxide (ITO)-coated glass (10 Ω, AMG Tech), ITO-coated poly(ethylene terephthalate) (PET (10 Ω, WJ Chemical) and Surlyn (60 µm, SOLARONIX). Conventional polypropylene (PP) nonwoven fabric was used. Polyurethane was purchased from Hepce Chemical, and silver-coated polyamide/polyester hybrid thread (85 Ω/m, Silver-tech) was used as a conductive thread.

### 2.2. Preparation of Electrochromic Films

Nonporous films of WO_3_ or WO_3_@PEO film were prepared by spin coating (5000 rpm for 30 s) on a pre-cleaned ITO glass (acetone-2-propanol-DI water). After spin coating, the sample was annealed at 135 °C for 3 min. For a porous WO_3_@PEO film, the dispersion of WO_3_ particles in ethanol (the amount of WO_3_ = 0.2 g) was mixed with PEO (*M*_w_ = 600,000) in different weight ratios, and anhydrous ethanol was added until the amount of PEO reached 10 wt% in the solution. The solution was stirred at 52 °C until it was homogeneously dispersed and was then cooled to ambient temperature. The fibrous and porous structure of WO_3_@PEO film was prepared by an electrospinning method. First, the prepared solution was loaded in a syringe, and the distance between the needle (27 G) and the collector was 10 cm. A pre-cleaned ITO glass was located on the collector and connected to the voltage power supplier (Bertan 230-01R, Spellman, Hauppauge, USA). The prepared solution was electrospun under the optimized conditions (the applied voltage was 20 kV, and the flow rate was 0.5 mL/h; temperature: 18–35 °C; humidity: 25–39%) and annealed at 135 °C for 3 min.

### 2.3. Fabrication of ECDs with Liquid Electrolytes

For the fabrication of the ECD, an electrolyte and another ITO glass were sequentially stacked. Surlyn was used as a separator. The liquid electrolyte was prepared with anhydrous PC with 0.5 M LiClO_4_, 0.05 M Fc, and 10 wt% PEO(*M*_w_ = 100,000) in the solution.

### 2.4. Preparation of an ECD with Gel Electrolytes on the Fabric

The fabric was coated with polyurethane to avoid electrolyte penetration. The conductive thread was patterned on the fabric to realize a conductive substrate. Subsequently, WO_3_@PEO fibers were directly electrospun on the fabric. The gel electrolyte and ITO-coated PET were sequentially stacked, and Surlyn was used as a separator. The gel electrolyte was prepared with anhydrous PC with 0.5 M LiClO_4_ and 0.05 M Fc, and the controlled amount of PEO (*M*_w_ = 100,000).

### 2.5. Characterizations

The morphology and thickness were characterized by scanning electron microscopy (FE-SEM; JSM-7610F, JEOL Ltd., Tokyo, Japan). Transmittance was measured with a UV-vis spectrometer (Lambda 35, PerkinElmer), and a voltage was applied using a potentiostat (IviumStat.h, HS Technologies, Gunpo, Korea). To obtain cyclic voltammetry (CV) curves, a three-electrode system was used. The electrochromic layer was used as the working electrode, and an Ag/AgCl electrode and ITO glass were employed as the reference electrode and counter electrode, respectively. The potential window of CV was −2.0 to 1.0 V, and different scan rates were employed.

## 3. Results and Discussion

### 3.1. Electrochromic Characteristics of Nonporous and Porous Films of WO_3_@PEO

**Figure 2:**
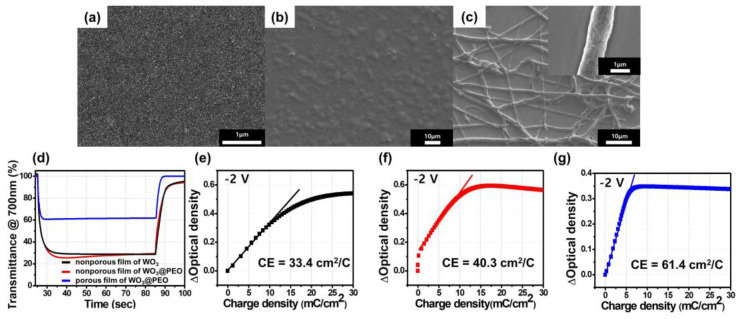
Scanning electron microscopy images of (**a**) a nonporous film of WO_3_; (**b**) a nonporous film of WO_3_@PEO with WO_3_:PEO = 1:1; (**c**) a porous film of WO_3_@PEO with WO_3_:PEO = 1:1; (**d**–**g**) electrochromic characteristics of a nonporous film of WO_3_ (black), a nonporous film of WO_3_@PEO with WO_3_:PEO = 1:1 (red), a porous film of WO_3_@PEO fibers with WO_3_:PEO = 1:1 (blue); (**d**) transmittance profiles at 700nm wavelength; (**e**–**g**) optical density of an ECD plotted against the charge density. The applied voltage was −2 and 1 V in the colored and bleached states, respectively.

To examine the effect of PEO-embedded fibers in a porous structure, we fabricated a WO_3_@PEO fibrous and porous structure prepared with WO_3_:PEO = 1:1 (*w*/*w*). Its electrochromic performance was compared with those of a nonporous WO_3_ film and a nonporous WO_3_@PEO film with WO_3_:PEO = 1:1 (*w*/*w*) (Figure 2). Their electrochromic characteristics with different composites and morphologies are summarized in Table 1. Here, η is the coloration efficiency, and *η*_/m_ is the normalized value of η with *m*, which is the weight of WO_3_ corresponding to the active area. The coloration time means a response time to achieve 90% of the entire transmittance change at a colored state, and Δ*T* is a transmittance difference between the bleached state (*T*_b_) and the colored state (*T*_c_) (ΔT = *T*_b_ − *T*_c_).

The coloration efficiency (η) was calculated as follows:

(2) $$\eta =log\left(\frac{T_{c}}{T_{b}}\right)/\Delta Q=\frac{\Delta OD}{\Delta Q}\;,$$

Here, ΔOD is the optical density change, and Δ*Q* is the change in the charge density corresponding to ΔOD [14]. η indicates the optical density change (ΔOD) for a change in the charge density (Δ*Q*) in a specific area [25]. The η of the nonporous film of WO_3_ was found to be 33.4 cm^2^/C when the applied voltage was −2 V (Table 1). On the other hand, the nonporous film of WO_3_@PEO with WO_3_:PEO = 1:1 showed a higher η of 40.3 cm^2^/C while ΔOD was similar to that of the nonporous film of WO_3_. This similarity was because a similar OD change occurred during the smaller Δ*Q* owing to the fast ion transport capability of PEO. PEO has been widely used as a solid electrolyte with high chain flexibility for promoting rapid ion transport. A large amount of Li^+^ donors present in ethylene oxide units can break/form Li-oxygen bonds and thereby promote Li-ion transports [26].

Next, to determine the effect of the porous structure of WO_3_@PEO, a porous film of WO_3_@PEO fibers with WO_3_:PEO = 1:1 was prepared through electrospinning, and its η characteristics were observed (Figure 2g). The Δ*Q* of the porous film of WO_3_@PEO was decreased more than that of the nonporous film of WO_3_@PEO by 50%, and it showed a larger η of 61.4 cm^2^/C, which was 184% larger than that of the nonporous film of WO_3_. An ideal electrochromic device is expected to exhibit high coloration efficiency [18]. The higher coloration efficiency of the porous film of WO_3_@PEO was obtained by a ΔOD with a smaller charge (Δ*Q*), as defined in Equation (2). The larger η resulted from the large surface area associated with the porous structure, providing a larger number of active sites than the nonporous film of WO_3_. Furthermore, most WO_3_ particles reacted quickly because of the short diffusion path of Li-ions in the porous structure [4].

### 3.2. Electrochromic Characteristics of the Porous Film of WO_3_@PEO Fibers Based ECD

**Figure 3:**
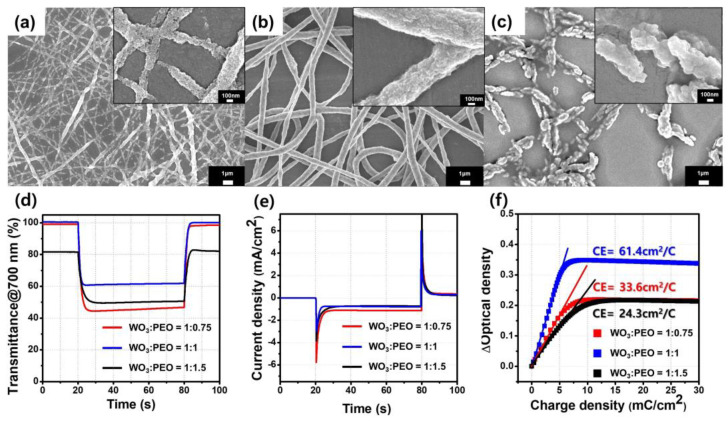
Scanning electron microscopy images of a porous film of WO_3_@PEO fibers with WO_3_:PEO ratios of (**a**) 1:0.75; (**b**) 1:1; (**c**) 1:1.5; (**d**,**e**) electrochromic characteristics of ECDs based on a porous film of WO_3_@PEO fibers with WO_3_:PEO ratios of 1:0.75 (red), 1:1 (blue), and 1:1.5 (black); (**d**) transmittance profiles at 700 nm wavelength; (**e**) current density plotted against time; (**f**) optical density plotted against charge density are also shown. The applied voltage was −2 and 1 V in the colored and bleached states, respectively.

We fabricated a porous film of WO_3_@PEO fibers with different WO_3_-PEO weight ratios to optimize the morphology of fibers. Figure 3a–c shows SEM images depicting the morphology of fibers. When the weight composition of PEO was 75% lower than that of WO_3_, an electrospinning solution was electro-sprayed because of its low viscosity. Therefore, the minimum ratio for WO_3_@PEO was 1:0.75 (WO_3_:PEO, *w*/*w*) (Figure 3a), and it was possible to form relatively uniform fibers despite the low PEO content. However, since PEO serves as a matrix for electrospinning to promote the formation of a fiber structure, the porous film of WO_3_@PEO fibers with WO_3_:PEO = 1:0.75 was not continuous, and its thickness was also not uniform. The average diameter value of fibers was about 115 nm. The porous film of WO_3_@PEO fibers with WO_3_:PEO = 1:1 (Figure 3b) was the most uniform fibrous structure, and its average diameter value of fibers was about 658 nm. In the case of the porous film of WO_3_@PEO fibers with WO_3_:PEO = 1:1.5 (Figure 3c), which had a higher PEO content, a large amount of PEO locally melted during the annealing process, and the fibrous structure could not be maintained. Thus, electro-spinnable conditions for the porous film of WO_3_@PEO fibers existed only at the WO_3_:PEO ratios of 1:0.75 and 1:1.

To analyze the electrochromic characteristics, we performed chronoamperometry (CA) and transmittance (*T,* in percentage). Based on these results, the η value was fitted. Figure 3d–f and Table 2 show the electrochromic characteristics of the ECDs as a function of the composition of PEO. Figure 3d shows Δ*T*-time plots, and Figure 3e depicts current density-time plots. The porous film of WO_3_@PEO fibers with WO_3_:PEO = 1:1 showed 40% Δ*T* for 1.6 s coloration time when the applied voltage was −2 V, and the η value was 61.4 cm^2^/C. The porous film of WO_3_@PEO fibers with WO_3_:PEO = 1:1.5, with a higher PEO amount, showed 54% of Δ*T* during the long coloration time of 3 s. Hence, the η was obtained as 24.3 cm^2^/C from the ΔOD-Δ*Q* graph in Figure 3f.

As shown in Figure 3c, for the porous film of WO_3_@PEO fibers with WO_3_:PEO = 1:1.5, the fibrous structure could not be maintained because of the low WO_3_ content after annealing, and a discontinuous structure was observed. Furthermore, WO_3_ particles were embedded in PEO fibers. The thicker PEO layer caused an increase in the diffusion distance of Li-ions, and the coloration time increased by three times over that of WO_3_:PEO = 1:1. Even at a Δ*T*, that was smaller by 32%, and a low η value of 24.3 cm^2^/C was observed. On the other hand, in the case of the porous film of WO_3_@PEO fibers with WO_3_:PEO = 1:0.75, which had a lower PEO content, the surface area was expected to be higher than that for WO_3_:PEO = 1:1 because of the thinner fiber [27]. Hence, Δ*T* was improved to 54% because of the larger surface area and higher WO_3_ contents compared with WO_3_:PEO = 1:1 (Table 2). However, since the amount of PEO, which promotes Li-ion transport, was small, the coloration time was 3.0 s, and the porous structure showed a η of 33.6 cm^2^/C, which was 55% smaller than the value for WO_3_:PEO = 1:1.

Thus, WO_3_:PEO = 1:1 was found to be an optimal ratio. At this point, we focused mainly on enhancing coloration time in electrochromic devices because most inorganic electrochromic materials have shown a few tens of seconds of coloration time [3,6]. This porous film of WO_3_@PEO showed the fastest coloration time compared with those previously reported (Table 3). When the η value for each ratio was normalized by the weight of WO_3_ (*η*_/m_), the porous film of WO_3_@PEO fibers with WO_3_:PEO = 1:1 showed a *η*_/m_ of 3610 cm^2^/mg·C. The reason for the high *η*_/m_ is that the porous structure provides many active sites in the same area, leading to high Δ*T* even for a small amount of WO_3_ particles.

To determine the characteristics corresponding to the optimal electrochromic layer thickness, the porous films of WO_3_@PEO fibers (WO_3_:PEO = 1:1) with different weights of WO_3_ were compared (Figure S1). As the amount of WO_3_ increased, the film thickness was thicker and fibers accumulated in multiple layers, causing reduced initial transmittance because of light scattering. Eventually, the overall Δ*T* decreased. The coloration time also became shorter because of the thicker electrochromic layer. The optimized layer thickness was determined to be 17 µm; at this thickness, the highest η and fastest coloration time were observed (Table S1).

**Figure 4:**
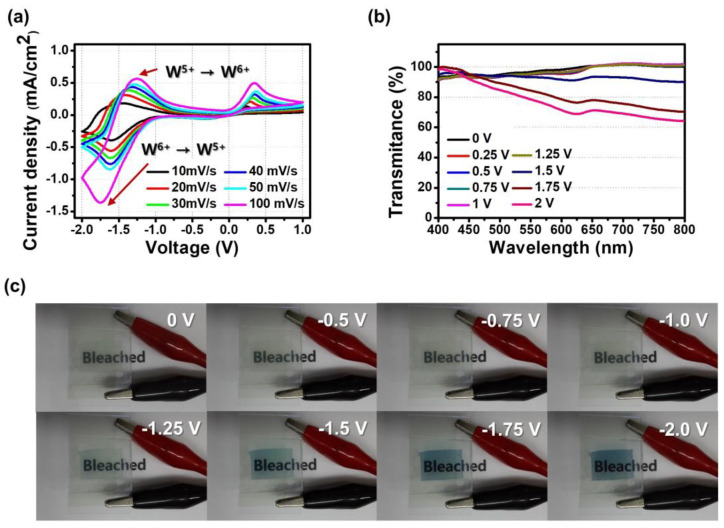
Electrochromic characteristics of a porous film of WO_3_@PEO fibers-based ECD with WO_3_:PEO = 1:1. (**a**) CV curves for different scan rates; (**b**) transmittance spectra for different applied voltages; (**c**) photographs of the ECD at differential voltage steps.

Figure 4a shows CV plots for various scan rates (10–100 mV/s). In the case of the CV curve at 10mV of scan rate, a reduction peak was observed at −1.75 V, where W^6+^ was reduced to W^5+^. Oxidation peaks were seen at −1.25 and 0.34 V. At −1.25 V, W^5+^ was oxidized to W^6+^, and the oxidation peak at 0.34 V is that of ferrocene (Fc) [31]. Fc is a counter redox material in the ECD. By this reaction, Fc introduced an alternative path for an electron to move, reducing the coloration time and reduction voltage [1,8]. Also, we found that as the scan rate increased, the difference between redox peaks was larger; however, it resulted in a higher current density due to a decrease in the size of the diffusion layer [32,33]. It can be inferred that the electrochemical reaction of ECD was stable at a slow scan rate below 100 mV/s.

The electrochemical characteristics from CV curves were consistent with the behavior of transmittance-wavelength plots for different applied voltages (Figure 4b). The porous film of WO_3_@PEO fiber-based ECD did not show significant Δ*T* for the voltage range of 0 V to −1.25 V. The transmittance started to decrease at −1.5 V and showed the minimum value at −2 V. This tendency is consistent with the gradual increase in the current density when the voltage was increased to −2 V in the CV curve. Figure 4c shows a photograph of the ECD for each applied voltage. It can be visually confirmed that the electrochromic layer was colored because of the reduction of WO_3_ when voltage was applied up to −1.5 V.

**Figure 5:**
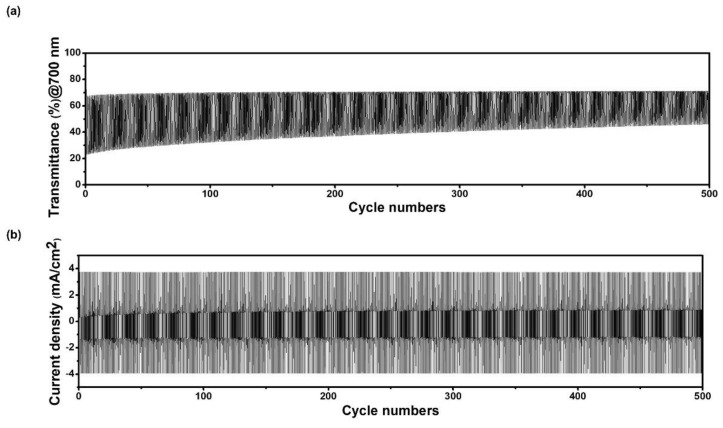
The cycle stability test for the ECD containing a porous film of WO_3_@PEO fibers with WO_3_:PEO = 1:1. (**a**) transmittance-cycle and (**b**) current density-cycle plots. A voltage was applied during the cycling test, and it was −2 V for the colored state and 1 V for the bleached state. Each voltage was applied for 10 s.

The cycle stability of a porous film of WO_3_@PEO-fiber-based ECD was measured using chronoamperometry (CA). Figure 5a shows the transmittance-cycle graph obtained by repeating the applied voltage from −2 V to 1 V and measuring the transmittance for 500 cycles. In Figure 5a, %*T* at the bleached state doesn’t show significant degradation until the 500th cycle, but %*T* at the colored state changes slightly. Hence, Δ*T* at the 250th cycle remained at 70.8% relative to the initial state, and there was no significant difference up to the 500th cycle. In the current density-cycle graph (Figure 5b), the current density was kept at a similar level during 500 cycles. It was confirmed that the porous film of WO_3_@PEO-fiber-based ECD worked reliably until 500 cycles.

### 3.3. Demonstration of an ECD on the Fabric

**Figure 6:**
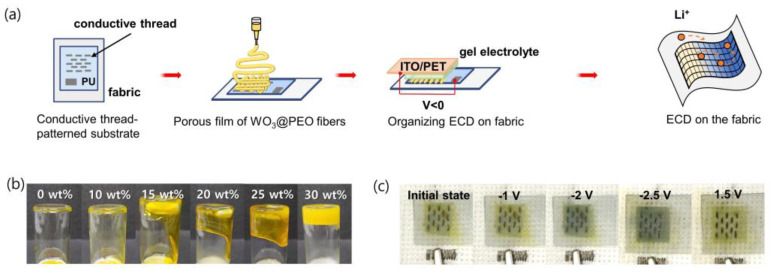
(**a**) Schematic image for fabrication of an ECD on the fabric; (**b**) photographs of the gel-electrolytes for different amounts of PEO in the solution; (**c**) photographs of ECD on fabric at different voltage steps.

Using the optimized porous film of WO_3_@PEO fibers discussed above, we fabricated an ECD on a flexible fabric rather than a rigid glass substrate (Figure 6a). To prevent hazards such as electrolyte leakage, a gel-type solid electrolyte was used instead of a conventional liquid electrolyte. The viscosity of a gel-type electrolyte controlled the amount of PEO (Figure 6b). When the amount of PEO was increased, the viscosity was increased, but the excessive PEO interrupted Li-ion transport and resulted in a higher reduction voltage and a side reaction. In the electrolyte with 30 wt% PEO, the ECD did not show any color change, even at −3 V. Hence, 25 wt% was considered the optimal amount of PEO. The fabricated ECD on fabric showed a comparable color change and operated at −2.5 V to 1.5 V of the voltage range (Figure 6c). The ECD on the fabric was fabricated through a simple process, which is advantageous for their economic feasibility and popularity. They can also be applied to wearable camouflage materials because they need excellent stability, even during intense activities.

## 4. Conclusions

In this work, we fabricated a porous structure of WO_3_@ PEO fibers using electrospinning. For achieving optimized electrochromic properties, the ratio of WO_3_ to PEO was adjusted. ECDs containing a porous film of WO_3_@PEO fibers with WO_3_:PEO = 1:1 showed the highest η, 61.3 cm^2^/C, along with a fact coloration time (1.6 s) and good cycle stability. Compared with a nonporous film of WO_3_@PEO, a porous film of WO_3_@PEO fibers in our study showed a fast coloration time because of the presence of many active sites on the porous electrochromic layer and the short diffusion path of Li-ions. Furthermore, it was shown that PEO, a promoter of Li-ion transport, promoted Li-ion transport and contributed to a fast coloration time. Additionally, we successfully fabricated ECD on a fabric by using an optimum composite of a porous film of WO_3_@PEO fibers and a gel electrolyte.

## Figures and Tables

**Table 1:** Electrochromic characteristics of WO_3_-based ECDs with different composites and morphologies.

	*η* (cm^2^/C)	*η*_/m_ (cm^2^/mg C)	Coloration Time (%90) (s)	Δ*T* (%)	WO_3_ (mg)
A nonporous WO_3_ film	22.3	69.7	4.0	73	0.32
A nonporous film of WO_3_:PEO (1:1, *w*/*w*)	41.2	258.0	4.7	74	0.16
A porous film of WO_3_:PEO fibers (1:1, *w*/*w*)	47.2	2770	1.6	40	0.017

**Table 2:** Electrochromic characteristics of the Porous Film of WO_3_@PEO-fiber-based ECD for different WO_3_:PEO ratios.

WO_3_: PEO (*w*/*w*)	Fiber Thickness (nm)	*η* (cm^2^/C)	*η*_/m_ (cm^2^/mg C)	Coloration Time (%90) (s)	Δ*T* (%)
1: 0.75	115 ± 60	33.6	259	3.0	54
1: 1	658 ± 54	61.4	3610	1.6	40
1:1.5	952 ± 192	24.3	101	4.5	32

**Table 3:** Comparison of electrochromic characteristics of previously reported WO_3_-based ECDs with an ECD fabricated in the present study.

	Coloration Time (s)	Applied Voltage (V)	*η* (cm^2^/C)	Δ*T*(%)	Ref.
WO_3_ microparticle film	12.5	−0.9–0	58.2	76.1	[14]
WO_3_ nanoparticle film	10	−1.5–0	34.3	52	[20]
Mesoporous WO_3_ film	2.4	−0.6–0.6	79.7	75.6	[28]
Amorphous WO_3_ film	17	−2.5–2.5	63	45.3	[29]
WO_3_ dispersed film	15	−0.9–0.9	62.1	77.8	[30]
a porous film of WO_3_@PEO fibers	1.6	−2–1	61.4	39.5	This work

## Data Availability

Not applicable.

## Supplementary Materials

The following supporting information can be downloaded at https://www.mdpi.com/article/10.3390/bios13020194/s1. Figure S1: Electrochromic characteristics of an ECD containing a porous film of WO_3_@PEO fibers with WO_3_:PEO = 1:1 for different weights of WO_3_.; Table S1: Electrochromic characteristics of an ECD containing a porous film of WO_3_@PEO fibers with WO_3_:PEO = 1:1 for different weights of WO_3_.
