# Supplementary material for: Enhanced Coloration Time of Electrochromic Device Using Integrated WO3@PEO Electrodes for Wearable Devices
Source: Biosensors (Basel). 2023 Jan 28;13(2):194. doi: 10.3390/bios13020194 (PMC9953346; doi:10.3390/bios13020194)
Supplement: Supplementary file 1 [file biosensors-13-00194-s001.zip › biosensors-2100221-supplementary.pdf]

biosensors

MDPI

Supplementary Materials

Enhanced Coloration Time of Electrochromic Device Using Integrated WO_3@PEO Electrodes for Wearable Devices

Haneul Kwon [1,†], Soohyun Kim [1,2,†], Mirim Ham [1], Yewon Park [1], Haekyoung Kim [2,*], Wonmok Lee [3,*], and Hyunjung Lee [1,*]

* Correspondence: hkkim@ynu.ac.kr (H.K.); wonmoklee@sejong.ac.kr (W.L.); hyunjung@kookmin.ac.kr (H.L.)
† These authors contributed equally to this work.

Table of contents

Figure S1. Electrochromic characteristics of an ECD containing a porous film of WO_3@PEO fibers with WO_3:PEO = 1:1 for different weights of WO_3. The different WO_3 weights considered were 17 (which is the optimal value, blue), 52 (black), 67 (red), and 69 μg (green): (a) transmittance versus time, (b) current density versus time, and (c) optical density versus charge density. The applied voltage was -2 and 1 V at the colored and bleached states, respectively……………………………………………………………2

Table S1. Electrochromic characteristics of an ECD containing a porous film of WO_3@PEO fibers with WO_3:PEO = 1:1 for different weights of WO_3. The applied voltage was -2 and 1 V at the colored and bleached states, respectively……………………………………………………….....3

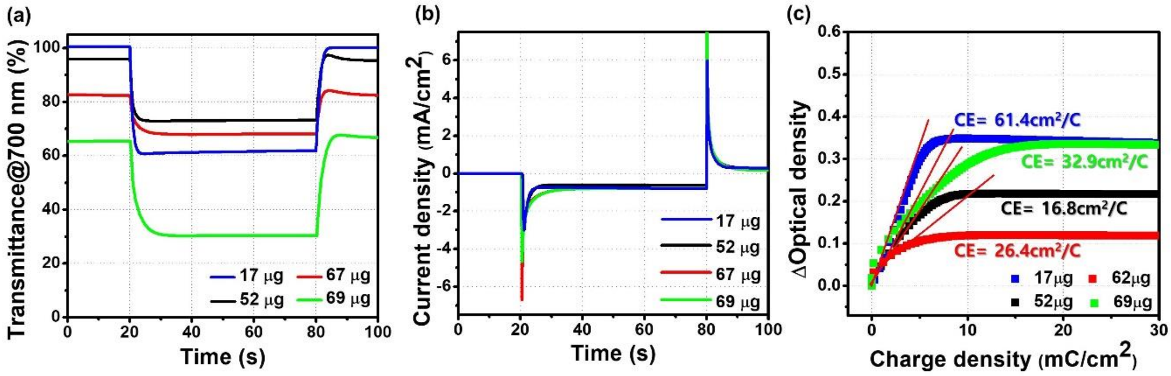

Figure S1. Electrochromic characteristics of an ECD containing a porous film of WO_3@PEO fibers with WO_3:PEO = 1:1 for different weights of WO_3. The different WO_3 weights considered were 17 (which is the optimal value, blue), 52 (black), 67 (red), and 69 μg (green): (a) transmittance versus time, (b) current density versus time, and (c) optical density versus charge density. The applied voltage was -2 and 1 V at the colored and bleached states, respectively.

Table S1. Electrochromic characteristics of an ECD containing a porous film of WO_3@PEO fibers with WO_3:PEO = 1:1 for different weights of WO_3. The applied voltage was -2 and 1 V at the colored and bleached states, respectively.

WO_3(μg)	η (cm^2/C)	$\eta_{/m}$(cm^2/mg · C)	Coloration time$_{(\%90)}$ (s)	ΔT (%)
17	61.4	3610	1.6	40
52	32.9	633	1.9	23
67	16.8	251	4	14
69	26.4	383	4.7	34
